# Human *Pasteurella multocida* Infection with Likely Zoonotic Transmission from a Pet Dog, Spain

**DOI:** 10.3201/eid2406.171998

**Published:** 2018-06

**Authors:** Fátima Abreu, Carlos Rodríguez-Lucas, M. Rosario Rodicio, Ana I. Vela, José Francisco Fernández-Garayzábal, Pilar S. Leiva, Fernando Cuesta, Dolores Cid, Javier Fernández

**Affiliations:** Principado de Asturias Sanitary Research Institute, Oviedo, Spain (F. Abreu, C. Rodriguez-Lucas, M.R. Rodicio, P.S. Leiva, J. Fernández);; Central University Hospital, Asturias, Spain (F. Abreu, C. Rodriguez-Lucas, P.S. Leiva, J. Fernández);; University of Oviedo, Asturias (C. Rodriguez-Lucas, M.R. Rodicio);; Complutense University, Madrid, Spain (A.I. Vela, J.F. Fernández-Garayzábal, D. Cid);; Principado de Asturias Health Care System, Pola de Siero Primary Care Center, Pola de Siero, Spain (F. Cuesta)

**Keywords:** *Pasteurella multocida*, urinary tract infection, UTI, pets, PFGE, MLST, molecular characterization, zoonoses, bacteria, bacterial infections, dog, Spain

## Abstract

We report a case of urinary tract infection caused by an unusual genotype (sequence type 211) of *Pasteurella multocida* associated with human infection. Molecular genetic analysis of *P. multocida* isolates obtained from the human patient and his pet strongly suggests a zoonotic transmission of this bacterium.

The bacterium *Pasteurella multocida* is one of the most frequent commensal and opportunistic pathogens found in domestic and wild animals worldwide (*1*). *P. multocida* is commonly cultured from the oropharynx of cats and dogs, and most human infections are associated with animal exposure, mainly from cats and dogs, and usually involve soft-tissue sites after animal bites or scratches (*1*). Among the wide clinical spectrum of invasive and noninvasive infections caused by *P. multocida*, urinary tract infections (UTIs) are rarely diagnosed, with <20 cases reported in the literature, most related to underlying diseases or urologic abnormalities (*2**,**3*). Here we present a case of UTI caused by an unusual genotype of *P. multocida*.

An 83-year-old man was referred to a primary healthcare center with urinary complaints and fever without his general condition being impaired. The patient had previously had prostatic adenoma and inguinal hernia diagnosed. Since 2013, he had suffered recurrent UTIs, which were treated empirically with oral ciprofloxacin, resulting in favorable clinical progression. In the last episode, urine analysis revealed the presence of proteins, nitrites, blood (10–25 cells/×400 microscope field), and abundant leukocytes (>100 cells/×400 microscope field). We sent a urine sample to the clinical microbiology laboratory of Hospital Universitario Central de Asturias and cultured in BBD CHROMagar Orientation Medium (Becton Dickinson, Heidelberg, Germany). We recovered bacterial counts (>10^5^ CFU/mL) of an oxidase-positive gram-negative coccobacillus producing small (≈1 mm) white colonies in pure culture. Matrix-assisted laser desorption/ionization time-of-flight mass spectrometry (Microflex; Bruker Daltonik GmbH, Bremen, Germany) identified the bacterium as *P. multocida* (score >2), and this finding was confirmed by 2-strand sequencing of the 16S ribosomal RNA gene (*4*). We performed antimicrobial drug susceptibility testing by using the NegCombo Type 44 MicroScan panel (Beckman Coulter, Brea, CA, USA) and interpreted the results according to Clinical and Laboratory Standards Institute guidelines (*5*). The isolate was susceptible to all antimicrobial drugs tested (β-lactams, β-lactams plus β-lactamase inhibitors, quinolones, colistin, tetracycline, tigecycline, chloramphenicol, trimethoprim/sulfamethoxazole, fosfomycin, and nitrofurantoin) except aminoglycosides. We administered oral ciprofloxacin (500 mg every 12 h for 1 wk) to the patient, who had an excellent outcome, including bacteriuria eradication.

Further questioning of the patient indicated that he had a dog at home. We placed gingival swabs obtained from the animal in Amies transport medium and sent them to the hospital’s clinical microbiology laboratory, where *P. multocida* was recovered. The animal isolate exhibited an antimicrobial drug susceptibility pattern identical to that of the patient isolate. Links between *P. multocida* human infections and pets are, in most cases, based on the information given by the patients indicating they have dogs or cats at home, but molecular studies aimed to associate *P. multocida* human infections with animal sources have rarely been conducted (*2**,**6*). To determine the source of the UTI, we subjected the patient and dog isolates to molecular typing. We determined the capsular types and genetically characterized the isolates by using multilocus sequence typing (*7*) and pulsed-field gel electrophoresis with *Apa*I and *Sma*I restriction enzymes (*8**,**9*). Both isolates belonged to capsular type A and to sequence type 211, a genotype that has been previously isolated only from avian wound infections (https://pubmlst.org/pmultocida). Moreover, both isolates exhibited indistinguishable pulsotypes with the 2 enzymes used (Figure). These facts, together with the identical antimicrobial drug susceptibility pattern, demonstrate that they are the same strain, thus establishing a definitive epidemiologic link between the patient and his dog.

**Figure:**
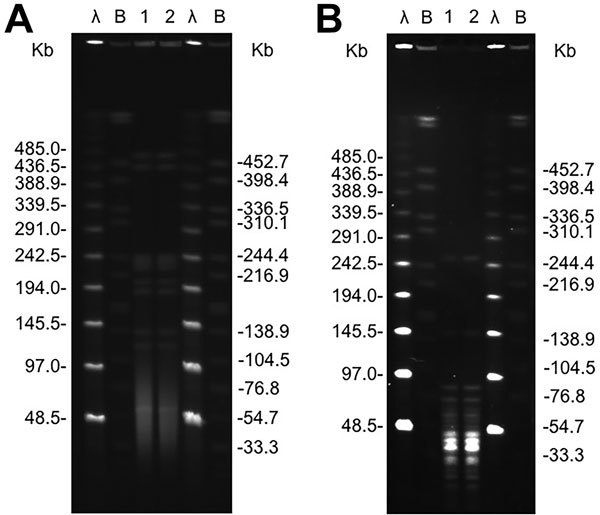
Pulsed-field gel electrophoresis profiles of *Apa*I (A) and *Sma*I (B) digested genomic DNA of *Pasteurella multocida* isolates from an 83-year-old man with a urinary tract infection (lane 1) and his pet dog (lane 2). Lanes λ (Lambda Ladder PFGE marker [New England BioLabs, Ipswich, MA, USA]) and lanes B (DNA from *Salmonella enterica* serovar Branderup H9812 digested with *Xba*I) used as molecular size standards.

The patient denied any history of recent bites or scratches, but *P. multocida* infections without a bite history can occur in patients with certain co-occurring conditions (*10*). The patient in this case had several predisposing factors, including a prostatic adenoma, which might have favored the infection by *P. multocida* because of mechanical alteration of the urinary tract. However, the specific route by which *P. multocida* reached the bladder could not be established. Although the possibility of a small scratch that had gone unnoticed cannot be ruled out, an alternative explanation could be a periurethral contamination of the patient after handling his dog, followed by colonization of the urethra and subsequent migration of the bacteria to the bladder. Although the specific route of transmission could not be elucidated in this case, pet owners and physicians should keep in mind that companion animals could be the source of infection by a wide range of opportunistic pathogens.
